# Effects of intrauterine growth restriction during late pregnancy on the cell growth, proliferation, and differentiation in ovine fetal thymuses

**DOI:** 10.5713/ab.21.0414

**Published:** 2022-01-21

**Authors:** Yang Zi, Chi Ma, Shan He, Huan Yang, Min Zhang, Feng Gao, Yingchun Liu

**Affiliations:** 1 College of Animal Science, Animal Nutrition and Feed Science at Universities of Inner Mongolia Autonomous Region, Inner Mongolia Agricultural University, Hohhot, 010018, China; 2 College of Life Science, Inner Mongolia Key Laboratory of Biomanufacturing, Inner Mongolia Agricultural University, Hohhot, 010018, China

**Keywords:** Cell Cycle Arrest, Cell Differentiation, Maternal Undernutrition, Ovine Fetal Thymus

## Abstract

**Objective:**

This study investigated the effects of intrauterine growth restriction (IUGR) during late pregnancy on the cell growth, proliferation, and differentiation in ovine fetal thymuses.

**Methods:**

Eighteen time-mated Mongolian ewes with singleton fetuses were allocated to three groups at d 90 of pregnancy: restricted group 1 (RG1, 0.18 MJ ME/body weight [BW]^0.75^/d, n = 6), restricted group 2 (RG2, 0.33 MJ ME/BW^0.75^/d, n = 6) and control group (CG, *ad libitum*, 0.67 MJ ME/BW^0.75^/d, n = 6). Fetuses were recovered at slaughter on d 140.

**Results:**

The G0/G1 phase cell number in fetal thymus of the RG1 group was increased but the proliferation index and the expression of proliferating cell nuclear antigen (PCNA) were reduced compared with the CG group (p<0.05). Fetuses in the RG1 group exhibited decreased growth hormone receptor (GHR), insulin-like growth factor 2 receptor (IGF-2R), and their mRNA expressions (p<0.05). For the RG2 fetuses, there were no differences in the proliferation index and PCNA expression (p>0.05), but growth hormone (GH) and the mRNA expression of GHR were lower than those of the CG group (p<0.05). The thymic mRNA expressions of cyclin-dependent protein kinases (CDKs including CDK1, CDK2, and CDK4), CCNE, E2-factors (E2F1, E2F2, and E2F5) were reduced in the RG1 and RG2 groups (p<0.05), and decreased mRNA expressions of E2F4, CCNA, CCNB, and CCND were occurred in the RG1 fetuses (p<0.05). The decreased E-cadherin (E-cad) as a marker for epithelial-mesenchymal transition (EMT) was found in the RG1 and RG2 groups (p< 0.05), but the OB-cadherin which is a marker for activated fibroblasts was increased in fetal thymus of the RG1 group (p<0.05).

**Conclusion:**

These results indicate that weakened GH/IGF signaling system repressed the cell cycle progression in G0/G1 phase in IUGR fetal thymus, but the switch from reduced E-cad to increased OB-cadherin suggests that transdifferentiation process of EMT associated with fibrogenesis was strengthened. The impaired cell growth, retarded proliferation and modified differentiation were responsible for impaired maturation of IUGR fetal thymus.

## INTRODUCTION

As one of the most important body’s immune organs, the thymus has long been known to be an organ that is vulnerable to atrophy, used as a sensitive index to detect their adverse effects on lymphocytes, when exposed to nutritional deficiencies [1,2]. The late fetal stage is a critical period of immune maturation [3], and a maturing and functional thymus is essential to the fetal immune system [4,5]. Intrauterine growth restriction (IUGR), resulting from maternal undernutrition which might be induced by inadequate food intake, poor nutritional quality of diets or imbalance nutrients of diets, has been associated with altered development of the fetal major organs [6–8], and the impaired growth of fetal thymus caused by maternal undernutrition has been observed [9–12]. Furthermore, the IUGR fetal thymus has a destroyed tridimensional structure, decreased ratio of cortical to medullary, fibrosis, antioxidant imbalance and dysfunction including decreased number of clusters of differentiation 3 positive (CD3^+^) T lymphocytes and T cell subpopulation [13,14]. The reduced cell proliferation and increased cell apoptosis are responsible for the impaired development and microenvironment of IUGR fetal thymus, and for partially modifying the maturation of CD4^+^CD8^+^ thymocytes [14]. However, the precise mechanisms responsible for programming fetal thymus growth, development, and differentiation in thymic mitogenic proliferation mediated by nutritional alterations remain unknown. As an experimental model of IUGR used extensively [15], therefore, the objective of this study was to investigate the effects of IUGR during late pregnancy on the cell growth, proliferation, and differentiation in ovine fetal thymuses.

## MATERIALS AND METHODS

### Animals and treatments

All experimental procedures were conducted in accordance with institutional guidelines for the care and use of laboratory animals in China [16]. This study is a companion study, and the details of animals, experimental design and detailed procedures have been presented previously [14]. Briefly, Eighteen Mongolian ewes carrying singletons were allocated to three groups at d 90 of gestation according to the metabolizable energy (ME): restricted group 1 (RG1, 0.18 MJ ME/body weight [BW]^0.75^/d, n = 6), restricted group 2 (RG2, 0.33 MJ ME/BW^0.75^/d, n = 6) and control group (CG, *ad libitum*, 0.67 MJ ME/BW^0.75^/d, n = 6). The ewes in their second or third parity were mated with rams at a synchronized estrus, after treatment for 12 days with CIDRs (EAZI-BREED CIDR, each containing 0.3 g progesterone in inert silicone elastomer, Pfizer Australia Pty Ltd., New Zealand) and an injection of pregnant mare serum gonadotropin (400 IU). Pregnancies were confirmed by ultrasound scanning at approximately d 50 of gestation (Medison-SA-600; Medison CO. LTD, Seoul, Korea), and had similar live weights (mean 52.82±2.67 kg) at d 90 of gestation. All animals were housed in individual pens, and chopped hay (about 10 cm, mainly *Leymus chinensis*) was supplied during late pregnancy. Since the fetus is considered to achieve 80% to 85% of its final birth weight during the last two months of gestation [6,17], maternal undernutrition was imposed from d 90 to d 140 of pregnancy. At the beginning of restriction, the ME and chemical composition in the hay were measured (Table 1), and then the daily intake of the hay offered in the RG1 and RG2 groups was calculated by the ewe body weight, nutrition value of hay, and the designed energy plane in the restricted groups. Restricted ewes were fed at 08:30 and 16:00 h each day, and the amount of feed offered was constant throughout the restriction period (Table 2). The ewes in the CG group were offered feed at 08:30, 11:00 and 16:00 h daily (the feed refusals were approximately 10% of the total amount offered). The animals had free access to water and mineral mixture blocks (containing per kilogram: Ca, 15 g; P, 11.5 g; Mg as MgSO_4_·H_2_O, 1 g; Fe as FeSO_4_·7H_2_O, 500 mg; Cu as CuSO_4_·5H_2_O, 250 mg; Zn as ZnSO_4_, 175 mg; Mn as MnSO_4_, 100 mg; Co as CoCl_2_·6H_2_O, 20 mg; I as KI, 40 mg; Se as Na_2_SeO_3_·5H_2_O, 1.5 mg; Yuantongweiye Co., Ltd., Inner Mongolian, China). All feed refusals were collected daily before feeding at 08:30, weighed and sub-sampled for chemical analysis.

### Slaughtering procedures

Detailed slaughter procedures were described previously [14,18,19]. Briefly, all fetuses were removed at 140 d of gestation, and fetal BW was recorded. Some of the thymic tissue was snap-frozen in liquid nitrogen and stored at −80°C. After rinsing with phosphate-buffered saline (PBS, pH 7.4), portions of the fetal thymus were immediately placed in paraformaldehyde fixative solution (0.1 mol/L, pH 7.4).

### Expression of proliferating cell nuclear antigen in fetal thymuses

After fixation for at least 2 days, the tissues were dehydrated and paraffin-embedded, sectioned at 4 to 6 μm and stained with immunohistochemistry commercial kits of proliferating cell nuclear antigen (PCNA, KGA320; KeyGEN Biotech, Nanjing, China) for microscopic examination. The paraffin-embedded tissue sections were dewaxed, rehydrated, and treated with antigen retrieval for 20 min in a microwave-oven. Sections were immersed in 3% hydrogen peroxide solution for 10 min to quench endogenous peroxidase activity. Non-specific binding was prevented by incubation with 10% normal goat serum for 10 min. The sections were incubated with mouse monoclonal anti-PCNA antibody for 1 h at 37°C and secondary antibody for 10 min at 37°C, then were visualized with diaminobenzidine (DAB) solution, lightly counterstained with hematoxylin. Each specimen was viewed under a standard microscope, and the total number of PCNA positive cells was counted with Image-Pro Express 6 software, in 6 random high-power fields (magnification×400), respectively. Data were expressed as the number of PCNA positive cells per field.

### Cell cycle and proliferation index in fetal thymuses

Portions of the freshly collected fetal thymus were washed with PBS (pH 7.4) and minced, then passed through a stainless-steel mesh (300 mesh). The thymic cell suspensions were centrifuged at 2,000 rpm per min for 10 min. Suspend approx. 1×10^6^ cells in 0.5 mL of PBS, and fix cells by adding 4.5 mL of pre-chilled cold 70% ethanol stored at 4°C for at least 2 h. Centrifuge 1 mL ethanol-suspended cells for 5 min at 1,200 rpm per min and decant ethanol thoroughly. After washing with PBS, the cell was suspended in 1 mL of propidium iodide staining solution (containing 0.5% propidium iodide, 0.25% TritonX-100 and 10 mg/mL Rnase; 4ABIO, Beijing, China) and incubated at room temperature for 30 min [20]. After washing and resuspending, the cells were analyzed on a flow cytometer (FACSCalibur; Becton Dickinson, Franklin Lakes, NJ, USA) and the G1-, S-, G2- and M-phase could be recognized in a proliferating cell population. Data were analyzed with CellQuest software (Becton Dickinson, USA). The proliferation index was calculated as the percentages of S, G2 and M phase cells occupying the different phases of the cell cycle.

### GH and IGF-2 in fetal thymuses

Growth hormone (GH) and insulin-like growth factor 2 (IGF-2) were measured by commercial kits (NJJCBIO, Nanjing, China). According to the manufacturer’s recommendations, approximately 0.5 g of the fetal thymus was rinsed and homogenized in 0.85% chilled normal saline to obtain a 10% thymus homogenate, then 50 μL aliquots of the supernatants were added to the microtiter plates coated by sheep antibodies of GH and IGF-2 labeled with horse radish peroxidase and incubated at 37°C for 30 min to become antibody-antigen-enzyme-antibody complex. After washing with PBS twice, 50 μL staining solution of tetramethyl benzidine was added. The reaction is terminated 15min later by a sulphuric acid solution, then determined at 450 nm in Microplate Reader (ELX800; BIO-TEKINSTUMENTS, Winooski, VT, USA).

### The mRNA expressions of GHR, IGF-2R, and cell cycle regulators in fetal thymuses

Total RNA from thymus was isolated using RNA extraction kits (TianGen, Beijing, China), and reverse-transcribed with a first-strand cDNA synthesis kit according to the manufacturer’s instructions (TransGen Biotech Co., Ltd, China). Using cDNA synthesized from 500 ng of total RNA as a template for one amplification, real-time reverse transcriptase (RT)-polymerase chain reaction (PCR) was performed using TransStart Green qPCR mix (TransGen Biotech Co., Ltd, China) in a Bio-Rad iQ 5 Real Time PCR System according to the instructions provided. The thermal cycling parameters were as follows: 95°C for 30 s for initial denaturation then 40 cycles of 95°C for 5 s, 60°C for 30 s and 72°C for 30 s. The sequences of gene-specific primers for real-time RT-PCR are listed in Table 3. All samples were measured in triplicate, and the real-time PCR assay had similar efficiency and within the range of 90% to 110%. The relative expression ratio of mRNA was calculated by R = 2−ΔΔCt [21]. Threshold cycle (Ct) was the value of PCR cycles at which the fluorescence signal of the PCR reaction reached a fixed threshold. For each sample, the Ct both for the target gene and endogenous control gene were determined to calculate ΔCt sample (Ct target gene – Ct endogenous control). In this study, the β-actin gene was used as an endogenous control. Subsequently, ΔΔCt (ΔCt sample – ΔCt CG) was determined, and the relative expression was calculated by 2−ΔΔCt. For the CG group, the ΔΔCt is equal to 0. Negative controls were performed in which cDNA was substituted by water.

### Markers of epithelial-mesenchymal transition in fetal thymuses

A 10% thymus homogenate was used for the measurement of E-cadherin (E-cad), OB-cadherin, and vimentin (VIM) in fetal thymuses according to the procedures of commercial kits (NJJCBIO, China). In the kits, purified sheep antibodies of E-cad, OB-cadherin, and VIM labeled with horse radish peroxidase are used to coat microtiter plate wells.

### Statistical analysis

All data were analyzed by using the analysis of variance procedure as implemented in SAS software [22]. Duncan’s test was used to identify significant differences between mean values. Significance was declared at p≤0.05.

## RESULTS

### Cell cycle, proliferation index and expression of proliferating cell nuclear antigen in ovine fetal thymuses

**Figure 1:**
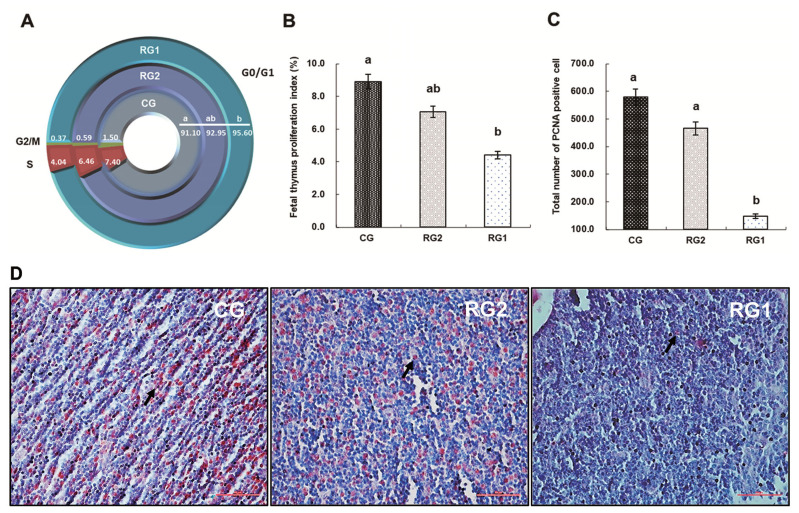
Effects of intrauterine growth restriction on the cell cycle, proliferation index and expression of proliferating cell nuclear antigen in ovine fetal thymuses during late pregnancy. Panel A shows the cell cycle in the fetal thymuses. Frames B shows the proliferation index in the fetal thymuses. Frames C shows the total number of PCNA positive cell in the fetal thymuses. Frames D from sections of fetal thymic tissues shows staining for proliferating cell nuclear antigen; Magnification, ×400 (the arrow indicates a red PCNA). Bars, 50 μm. CG, control group, *ad libitum*, 0.67 MJ ME/BW^0.75^/d; RG2, restricted group2, 0.33 MJ ME/BW^0.75^/d; RG1, restricted group1, 0.18 MJ ME/BW^0.75^/d; G0/G1, gap 0/1 phase; S, synthesis of DNA phase; G2/M, gap 2 phase/cell division phase. PCNA, proliferating cell nuclear antigen; ME, metabolizable energy; BW, body weight. Duncan’s test was used to identify significant differences between mean values. ^a,b^ Means without common letters are different at p<0.05.

The cell cycle, proliferation index and expression of PCNA in ovine fetal thymuses are summarized in Figure 1. With the reduction of maternal energy intake, the G0/G1 phase cell number in fetal thymus of the RG1 group was increased compared with the controls (p<0.05), but reduced PCNA expression and proliferation index were found in the RG1 fetal thymuses compared with the CG group (p<0.05). For the S phase and G2/M phase cell number in fetal thymuses, there were no differences between the restricted groups and the CG group (p>0.05).

### GH, IGF-2, and their receptors mRNA expressions in fetal thymuses

GH, IGF-2, and their receptors expressions in fetal thymuses are presented in Table 4. Fetuses in the RG1 group exhibited decreased thymic GH and IGF-2 (p<0.05), and the mRNA expressions of thymic growth hormone receptor (GHR) and IGF-2R were lower than those of the CG group (p<0.05). For the RG2 group, GH and GHR mRNA expression in fetal thymuses were decreased relative to the controls (p<0.05).

### The mRNA expressions of cell cycle regulators in fetal thymuses

**Figure 2:**
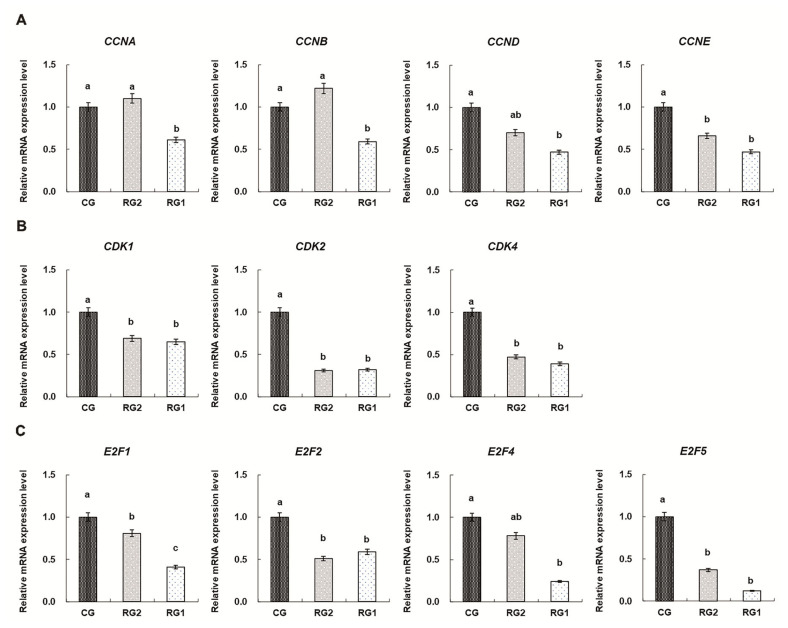
Effects of intrauterine growth restriction during late pregnancy on mRNA expressions of cell cycle regulators in ovine fetal thymuses. (A) Relative mRNA levels of cyclins. (B) Relative mRNA levels of cyclin-dependent protein kinases. (C) Relative mRNA levels of E2-factors. The real-time polymerase chain reaction assay had similar efficiency and within the range of 90% to 110% and the *ACTB* gene (β-actin) was used as an endogenous control. The relative expression ratio of mRNA was calculated by R = 2^−ΔΔCt^. CG, control group, *ad libitum*, 0.67 MJ ME/BW^0.75^/d; RG2, restricted group2, 0.33 MJ ME/BW^0.75^/d; RG1, restricted group1, 0.18 MJ ME/BW^0.75^/d; CCNA, cyclin A; CCNB, cyclin B; CCND, cyclin D; CCNE, cyclin E; CDK1, cyclin-dependent protein kinase 1; CDK2, cyclin-dependent protein kinase 2; CDK4, cyclin-dependent protein kinase 4; E2F1, E2-factor 1; E2F2, E2-factor 2; E2F4, E2-factor 4; E2F5, E2-factor 5; ME, metabolizable energy; BW, body weight. Duncan’s test was used to identify significant differences between mean values. ^a–c^ Means without common letters are different at p<0.05.

The mRNA expressions of cell cycle regulators in fetal thymuses are shown in Figure 2. Reduced thymic mRNA expressions of CDK1, CDK2, CDK4, CCNA, CCNB, CCND, and CCNE in the RG1 group were found compared with the controls (p<0.05). For the RG2 group, the mRNA expressions of CDK1, CDK2, CDK4, and CCNE were lower than those of the CG group (p<0.05). Thymic mRNA expressions of E2F1, E2F2, and E2F5 were reduced in the RG1 and RG2 groups (p<0.05) and reduced thymic E2F4 expression was found in the RG1 group (p = 0.0037).

### The markers expressions of epithelial-mesenchymal transition in fetal thymuses

**Figure 3:**
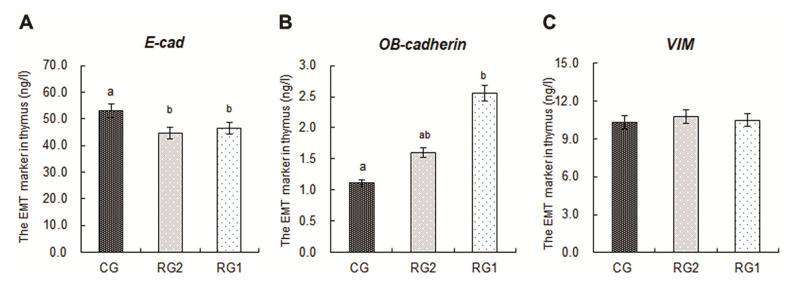
Effects of intrauterine growth restriction on the markers of epithelial-mesenchymal transition in ovine fetal thymuses during late pregnancy. CG, control group, *ad libitum*, 0.67 MJ ME/BW^0.75^/d; RG2, restricted group2, 0.33 MJ ME/BW^0.75^/d; RG1, restricted group1, 0.18 MJ ME/BW^0.75^/d; E-cad, E-cadherin; VIM, vimentin; ME, metabolizable energy; BW, body weight. Duncan’s test was used to identify significant differences between mean values. ^a,b^ Means without common letters are different at p<0.05.

The markers expressions of epithelial-mesenchymal transition (EMT) in fetal thymuses are presented in Figure 3. The decreased expressions of E-cad in the RG1 and RG2 groups were found relative to the controls (p<0.05), and the expression of OB-cadherin in the RG1 group was higher than that of the CG group (p<0.05). For the VIM expressions in fetal thymuses, there were no differences among the RG1, RG2 and CG groups (p>0.05).

## DISCUSSION

While the thymus involutes shortly after birth it continues to function well into adult life [23]. As a sensitive organ to nutrition, however, thymic atrophy is one of the most conspicuous changes resulting from impaired cell proliferation in malnourished rats and fetal sheep [14,24]. For cell proliferation, normal cell cycle is a crucial task for every multicellular organism because it determines size and shape, tissue renewal and senescence [25]. The eukaryotic cell cycle refers to the series of events comprising the sequential actions including synthesis of DNA (S-phase) and cell division (M-phase) with intervening gap phases to allow cell growth (G1-phase) and to check the integrity of genomic material (G2-phase) [26]. In the present study, decreased fetal thymic proliferation index in the RG1 group, expressed as the percentages of S, G2, and M phase cells occupying the different phases of the cell cycle, indicate that the cell cycle in fetal thymus was arrested at G0/G1 phase by maternal undernutrition, which was consistent with deceased PCNA expression in the RG1 fetal thymuses. At late G1 phase, a nutrient-sensitive cell growth checkpoint (labeled as R) controls progression to S phase, and enables the cell to complete cell division, but only when sufficient nutrients are available [27]. Because G1 progression requires a favorable metabolic state to accumulate enough cell mass and organelles for establishing two daughter cells [28], the control of cellular proliferation is achieved primarily in the G1-phase of the cell cycle in mammalian cells [29]. The ovine fetal thymic G0/G1 cell cycle arrest induced by maternal undernutrition was responsible for impaired cellular proliferation in IUGR fetal thymus, and led to IUGR fetal thymic growth retardation [14].

Progression through each cell cycle phases (G1, S, G2, and M) is under the control of protein kinases, which are heterodimers consisting of a catalytic subunit (the cyclin-dependent protein kinase, CDK) and a regulatory subunit (the cyclins) required for controlling CDK activity and substrate specificity [30]. Of these, CDK1, CDK2, and CDK4/6 and A, B, E, D-type cyclins are identified as the major regulators [31]. Activated CDKs, binding to their selected cyclins, phosphorylate their target proteins to control the cell cycle by maintaining exit and entry to different phases of the cell cycle [32]. Accordingly, CDKs are perceived as the engine that drives cell cycle progression and the expression of CDKs remains relatively constant throughout the cell cycle, whereas cyclins are synthesized and destroyed in a cyclical fashion during cell cycle and considered to be the gears that are changed to aid the transition between cycle phases [30,33]. The data in this study show that there were differences in CDKs expressions in fetal thymuses of both IUGR groups, and decreased expressions of CCNA, CCNB, CCND, and CCNE in the RG1 fetal thymuses were found. The results suggest that the expressions of CDKs as the engine drives driving the cell cycle are more sensitive to maternal undernutrition than the expressions of cyclin as the gears aiding the transition between cycle phases in the IUGR fetal thymus. However, suppressed cell cycle progression in G0/G1 phase in the RG1 fetal thymus depended on down-regulating expressions of both CDKs and cyclins. In addition, transcription factors of the E2-factor family are critical for G1-S progression and divided into “activators” (E2F1-3) and “repressors” (E2F4-8) [34]. Down-regulated expressions of E2Fs played important role in suppressing the transcriptions of CDKs and cyclins and inducing the G0/G1 cell cycle arrest in IUGR fetal thymus.

GH/IGF signaling system is a master regulator stimulat ing cellular and somatic growth in vertebrates [35]. As one of the pituitary peptides, GH improves thymic epithelial cells (TECs) growth and stimulates proliferation of thymocytes, and directly involves in intrathymic migration of thymocytes and their export to the periphery, which have profound effects on modulation of numerous thymic functions and regulation of the immune system [36,37]. Insulin-like growth factor 2 that is the dominant member of the insulin family expressed during fetal life by the thymic epithelium [38], rather than IGF-I or insulin, is an important tissue factor for thymopoiesis [39]. In terms of cell cycle regulation, GH-IGF system induces signaling pathways for cell growth that compete with other signaling systems that result in cell death, and the final effect of these opposed forces is critical for normal and abnormal cell growth [40]. Furthermore, the cell proliferation and T-cell maturation are inexorably linked [23]. In the present study, decreased GH and GHR in both IUGR groups and the reductions of IGF-2 and IGF-2R in the RG1 fetal thymus were found. In addition, the reductions of E-cad in fetal thymus of the RG1 and RG2 groups, a marker of epithelial cells [41], were observed. As the final effect of opposed forces which repressed the cell cycle progression in G0/G1 phase in the IUGR fetal thymus by down-regulating expressions of both CDKs and cyclins, weakened GH/IGF signaling system in the IUGR fetal thymus was critical for abnormal IUGR fetal thymic cell growth and thymopoiesis, and was responsible for the retardation of the TECs growth and maturation of thymocytes.

T cell development requires a complex microenviron menfuzet including endothelial, dendritic, and TECs [42]. TECs are the key components in thymic microenvironment for T cells development and highly proliferative during thymic expansion [42,43]. A decreased change in expression of E-cad is the epithelial cell marker of EMT, which is a fundamental biological process by which epithelial cells undergo biochemical shifts to become mesenchymal cells that have different polarization from the original epithelia [44,45]. In addition, because OB-cadherin is a more definitive marker for activated fibroblasts, an E-cad and OB-cadherin switch is of interest for EMT associated with fibrogenesis [46]. In this study, the reductions of fetal thymic E-cad in both the restricted groups and increasing of OB-cadherin in the RG1 group illustrate that the transdifferentiation process of EMT associated with fibrogenesis were strengthened in the IUGR fetal thymus by maternal undernutrition. During EMT, epithelial cells lose their junctions and apical-basal polarity, reorganize their cytoskeleton, undergo a change in the signaling programs that define cell shape, and reprogram gene expression, which increases the motility of individual cells and enables the development of an invasive mesenchymal phenotype [47]. The destroyed tridimensional structure, decreased ratio of cortical to medullary and fibrosis, impaired maturation of CD4^+^CD8^+^ thymocytes, increased cell apoptosis in IUGR fetal thymuses have been reported in our previous results [14]. The EMT in IUGR fetal thymus in this study led to retarded growth of the TECs, destroyed tridimensional structure of their junctions and fibrogenesis directly, which were responsible for impaired maturation of CD4^+^CD8^+^ thymocytes and increased cell apoptosis [14].

## IMPLICATIONS

In conclusion, our results demonstrate that the cell cycle in fetal thymus was arrested at G0/G1 phase by maternal undernutrition during late pregnancy, and down-regulated expressions of E2Fs, CDKs and cyclins played important roles in the impairment of cell cycle progression in IUGR fetal thymuses. Weakened GH/IGF signaling system in the IUGR fetal thymus was observed, but the transdifferentiation process of EMT associated with fibrogenesis was strengthened induced by maternal undernutrition. The impaired cell growth, retarded proliferation and modified difference were responsible for impaired maturation of CD4^+^CD8^+^ thymocytes and fetal immune system.

## Figures and Tables

**Table 1:** Composition of grass hay and refusals during the restriction period

Item	Grass hay	Refusals
ME (MJ/kg)	8.90	-
DM (%)	88.42	91.99
CP (%)	10.09	9.27
EE (%)	4.34	2.72
NDF (%)	71.98	71.19
ADF (%)	35.82	36.60
Ash (%)	4.67	4.39
Ca (%)	0.57	0.68
P (%)	0.09	0.08

ME, metabolisable energy; DM, dry matter; CP, crude protein; EE, ether extract; NDF, neutraldetergent fiber; ADF, acid detergent fiber; Ca, calcium; P, phosphorus.

**Table 2:** Maternal feed consumption in different groups during late pregnancy

Item	Treatments^1)^			SEM	p-value

	CG (*ad libitum*)	RG2	RG1		
Mean daily grass intake (g/d)	1,693^a^	853^b^	444^c^	8	<0.0001
Mean daily crude protein intake (g/d)	171^a^	86^b^	45^c^	1	<0.0001
Daily metabolizable energy intake (MJ ME /BW ^0.75^/d)	0.67	0.33	0.18		

SEM, standard error of the mean; ME, metabolizable energy; BW, body weight.

1) CG, control group, *ad libitum*; RG2, restricted group2; RG1, restricted group1.

a–c Within a row, means without a common superscript differ (p<0.05).

**Table 3:** Information on the primers used for quantitative real-time polymerase chain reaction

Gene	Primer sequence (5′-3′)	GenBank Accession NO.	Amplicon length (bp)
*GHR*	Forward:5′- TGTGAGAACCCGACAACGAAAC -3′	NM_001009323	155
	Reverse:5′- TCACTGTTAGCCCAAGTATTCC -3′		
*IGF-2R*	Forward:5′ - ATACGTGGTCTCGGGCATTG-3′	AF353513.1	287
	Reverse:5′- TCCCTGGGTAAACGTCGTCAT- 3′		
*CCNA*	Forward:5′-ACCATGAGGACATTCACACGTACC- 3′	NM_001075123.1	124
	Reverse:5′-ACTAACCAGTCCACGAGGATAGCC- 3′		
*CCNB*	Forward:5′-GGAAATGTACCCTCCAGAAATCG-3′	NM_001045872.1	232
	Reverse:5′-CATATCGTAGTCCAGCATAGTTAGTT-3′		
*CCND*	Forward:5′- ACATGGAGCTGGTCCTGGTGA -3′	HQ825087.1	188
	Reverse:5′- GGAGGGTGGGTTGGAAATGAA -3′		
*CCNE*	Forward:5′-GAGAAGCCAGTGTGGCAGTC -3′	XM_015100542.2	157
	Reverse:5′-CGACGCTCTGGATGACGATG-3′		
*Cdk1*	Forward:5′- GTCAAGTGGTAGCCATGAAGAA -3′	NM_001142508.1	218
	Reverse:5′- GAACTGACCAGGAGGGATAGAA -3′		
*Cdk2*	Forward:5′- ACAAGTTGACGGGAGAAGTG -3′	FJ422550.1	235
	Reverse:5′- AGAGGAATGCCAGTGAGTGC -3′		
*Cdk4*	Forward:5′- CAGTGGCTGAGATTGGTGTCG -3′	NM_001127269.1	148
	Reverse:5′- ACCTCCCGAACGGTGCTGAT -3′		
*E2F1*	Forward:5′- AGGTGCTGAAGGTGCAGAAACGG -3′	XM_004014812.1	283
	Reverse:5′-CGAAGGTCCTGGCAGGTCACATA -3′		
*E2F2*	Forward:5′-AAGCGACGCATCTACGACATCAC-3′	XM_027965537.1	169
	Reverse:5′-GCTCCATGCTCATCAGCTCCTTC-3′		
*E2F4*	Forward:5′-GGGTGCTAACAGGAAGAAATGGA -3′	XM_004015584.1	134
	Reverse:5′-GCAAATGGCTCTAAATGAGGGTAAAT -3′		
*E2F5*	Forward:5′-CTTCACATCCACCAACCCTCCAC-3′	XM_004012027.1	199
	Reverse:5′-GAACAGTCTTGCGGCAGTAAACG-3′		
*ACTB*	Forward:5′-TCTTCCAGCCGTCCTTCCT-3′	NM_001009784.3	144
	Reverse:5′-TGCCAGGGTACATGGTGGT-3′		

*GHR*, growth hormone receptor; *IGF-1R*, insulin-like growth factor 1 receptor; IGF-2R, insulin-like growth factor 2 receptor; *CCNA*, cyclin A; *CCNB*, cyclin B; *CCND*, cyclin D; *CCNE*, cyclin E; *CDK1*, cyclin-dependent protein kinase 1; *CDK2*, cyclin-dependent protein kinase 2; *CDK4*, cyclin-dependent protein kinase 4; *E2F1*, E2-factor 1; *E2F2*, E2-factor 2; *E2F4*, E2-factor 4; *E2F5*, E2-factor 5; *ACTB*, β-actin.

**Table 4:** Effects of intrauterine growth restriction on GH, IGF-2, and their receptors expressions in ovine fetal thymuses during late pregnancy

Item	Treatments^1)^			SEM	p-value

	CG (n = 6)	RG2 (n = 6)	RG1 (n = 6)		
GH (ng/mL)	2.77^a^	2.13^b^	1.92^b^	0.11	0.0023
IGF-2 (ng/mL)	76.50^a^	68.59^a^	58.61^b^	2.50	0.0057
mRNA expressions of receptors					
GHR	1.00^a^	0.53^b^	0.21^b^	0.12	0.0041
IGF-2R	1.00^a^	0.82^a^	0.42^b^	0.064	0.0006

SEM, standard error of the mean; GH, growth hormone; IGF-2, insulin-like growth factor 2; GHR, growth hormone receptor; IGF-2R, insulin-like growth factor 2 receptor; ME, metabolizable energy; BW, body weight.

1) CG, control group, *ad libitum*, 0.67 MJ ME/BW^0.75^/d; RG2, restricted group2, 0.33 MJ ME/BW^0.75^/d; RG1, restricted group1, 0.18 MJ ME/BW^0.75^/d.

a,b Within a row, means without a common superscript differ (p<0.05).
